# The relationship between a body shape index and abdominal aortic calcification: a population-based study

**DOI:** 10.1016/j.clinsp.2026.100910

**Published:** 2026-03-25

**Authors:** Youfu Wang, Yiming Su, Changzhi Luo, Han Yang, Xiao Qin

**Affiliations:** a Department of Vascular Surgery Ward, The Fourth Affiliated Hospital of Guangxi Medical University, Nanning, Guangxi, China; b Department of Vascular Surgery Ward, The First Affiliated Hospital of Guangxi Medical University, Nanning, Guangxi, China

**Keywords:** Cross-sectional research, Abdominal aortic calcification, Severe abdominal aortic calcification, A body shape index, NHANES

## Abstract

**Background:**

Abdominal Aortic Calcification (AAC) is closely associated with visceral adiposity. The authors aimed to investigate the relationship between a Body Shape Index (ABSI) and AAC.

**Methods:**

Data were obtained from the 2013‒2014 cycle of the National Health and Nutrition Examination Survey (NHANES). Weighted multivariable regression, tests for trends, subgroup analysis, and smooth curves were used to explore the association between ABSI and AAC scores, as well as Severe Abdominal Aortic Calcification (SAAC). Receiver Operating Characteristic (ROC) curves were plotted to compare the predictive abilities of ABSI, Weight-adjusted Waist Index (WWI), Body Mass Index (BMI), Waist-to-Height Ratio (WHtR), and Waist Circumference (WC) for AAC and SAAC.

**Results:**

A total of 2741 individuals were included in the present study. The weighted incidence of AAC was 29.12%, and SAAC was 7.94%. After fully adjusting for covariates, each one-unit increase in ABSI was associated with a 0.42-point increase in the AAC score (95% CI: 0.29, 0.63), and the risk of SAAC increased by 37% (95% CI: 1.12, 1.77). Smooth curves showed a nonlinear positive correlation between ABSI and AAC scores, as well as SAAC. No dependence was observed in the subgroup analysis. ROC analysis showed that ABSI was a stronger predictor than WWI, BMI, WHtR, and WC.

**Conclusions:**

ABSI has a positive correlation with AAC scores and SAAC. However, prospective studies are required to validate the present results.

## Highlights

• ABSI positively and nonlinearly associates with AAC and SAAC.
• ABSI outperforms BMI, WWI, WHtR and WC in predicting AAC/SAAC.
• The ABSI-AAC/SAAC association is consistent across subgroups.

## Introduction

Abdominal Aortic Calcification (AAC) is a pathological process in which minerals, such as calcium-phosphate complexes, are deposited in the vascular system, and vascular smooth muscle cells undergo osteogenic differentiation and mineralization in response to this stimulus.^1^^,^^2^ A cohort study indicated that the prevalence of AAC in males and females aged 55- to 64-years was 81.8 % and 5 9.1 %, respectively.^3^ Numerous factors contribute to AAC, such as dyslipidemia, diabetes, hormones, inflammatory factors, smoking, and old age.^4^^,^^5^ Obesity, especially abdominal obesity, is an important contributor to AAC. It was found that abdominal visceral adiposity content was significantly and positively correlated with AAC by computed tomography scanning studies (*p* = 0.006, α = 0.05). This relationship was still significant after adjusting for the effects of confounding variables, indicating that adiposity accumulation is an important factor in causing AAC.^6^ Jensky et al. demonstrated that an increase in the ratio of muscle tissue to fatty tissue reduced the incidence of AAC (OR = 0.7) and that fatty tissue was positively associated with prevalent aortic calcification.^7^ AAC, especially Severe Abdominal Aortic Calcification (SAAC), is a marker of vascular aging and cardiovascular diseases such as myocardial infarction, heart failure, and stroke.^5^ Epidemiologic studies have reported that SAAC can predict mortality in cardiovascular diseases.^8^

Although abdominal fatty accumulation is an important marker for predicting AAC, most of the current data on abdominal fat are obtained by computed tomography scanning, a method that is difficult to generalize because of its limitations, high cost, and radiation hazards to participants, especially pregnant women.^9^ A Body Shape Index (ABSI) is a new body index calculated by waist circumference, Body Mass Index (BMI), and height. High ABSI indicates that the waist circumference is higher than the standard height and weight, and compared to BMI, which is unable to differentiate between the accumulation of muscle and fat, ABSI is better able to reflect the accumulation of abdominal fat.^10^ It has been shown that ABSI has demonstrated an excellent ability to predict diabetes, cardiovascular events, frailty, and mortality in middle-aged and older adults,^11^, ^12^, ^13^, ^14^ suggesting that ABSI may serve as a simple, noninvasive marker of adverse vascular phenotype. Although previous studies have explored the relationship between ABSI and AAC, important limitations remain. In particular, some prior analyses did not account for the complex sampling design of the National Health and Nutrition Examination Survey (NHANES), including the use of sampling weights, which is essential for generating unbiased, nationally representative estimates in U.S. adults. In addition, it is still unclear whether ABSI is associated not only with the presence of AAC but also with the overall calcification burden (AAC score) and with SAAC, and how this association changes across increasing ABSI levels. Therefore, using data from the NHANES 2013‒2014 survey cycle, the present study aimed to evaluate the association between ABSI and abdominal aortic calcification in a nationally representative sample of U.S. adults, including AAC presence, AAC score as a measure of calcification burden, and SAAC, while accounting for the complex NHANES sampling design.

## Materials and methods

### Study population

**Fig. 1:**
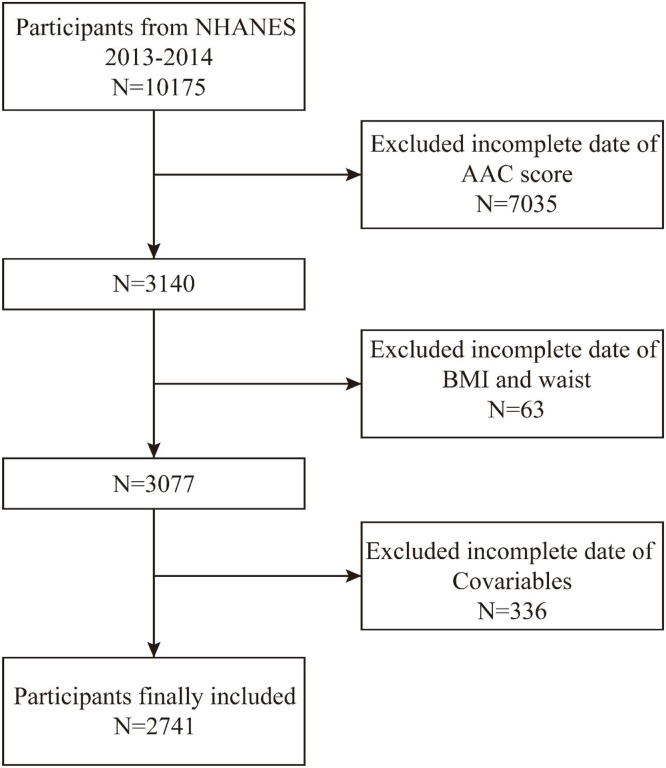
Flowchart of the study.

The National Health and Nutrition Examination Survey (NHANES) is administered by the National Center for Health Statistics (NCHS), and its Research Ethics Review Committee reviews and approves NHANES survey protocols (Protocol n° 2011–17). It is a nationally representative study that includes all age groups in the U.S.The data for this research were obtained from the NHANES 2013‒2014 survey cycle, which included 10,175 participants. After excluding participants with missing data ‒ 7035 with missing AAC scores, 63 with missing BMI and waist circumference, and 336 with missing covariates ‒ a total of 2741 participants were enrolled in this study (Fig. 1). This cross-sectional study follows the STROBE Statement.

### Evaluation of the AAC

NHANES performed spinal radiographs on people over 40-years of age and obtained AAC data from lateral spinal radiographs of the first through fourth lumbar vertebrae. The AAC was scored using the AAC-24 scoring semi-quantitative technique, with a total score ranging from 0 to 24. A score of 0 is no AAC; a score greater than 0 is considered AAC; and a score greater than 6 is considered SAAC.^15^ NCHS performs high-quality control of data collection and scan analysis. The ACC data collection process is described on the official NHANES website: https://www.cdc.gov/nchs/nhanes/.

### Assessment of ABSI

The ABSI was calculated as BMI, waist circumference, and height, with BMI calculated as weight in kilograms divided by height in meters squared, waist circumference measurements were localized by bilateral iliac crests, and height was the maximum vertical height while standing. All measurements were done by trained health technicians and record keepers. The ABSI was calculated as waist circumference / (BMI 2/3 × height 1/2).^11^

### Covariates

Based on previous studies, the covariates in this study were grouped into two categories. Demographic and lifestyle characteristics included age, gender, marital status, education level, race, alcohol consumption, and smoking. Clinical comorbidities and laboratory parameters included diabetes, hypertension, Coronary Heart Disease (CHD), stroke, cancer, arthritis, Chronic Obstructive Pulmonary Disease (COPD), liver condition, total calcium (mmoL/L), cholesterol (mmoL/L), triglycerides (mmoL/L), uric acid (µmoL/L), creatinine (µmoL/L), and 25-hydroxyvitamin-D3 (25(OH)D3) (nmoL/L). Detailed information on all covariates in this study can be accessed by visiting the NHANES database: https://www.cdc.gov/nchs/nhanes/.

### Statistical analyses

This study was statistically analyzed using R software (version 4.3) and EmpowerStats (version 4.2). ABSI was categorized into quartiles, and differences between ABSI quartiles for each variable were compared. Continuous variables and categorical variables were expressed as mean ± standard deviation and proportions, respectively. If the continuous variables were normally distributed, *t*-tests were used; otherwise, Mann-Whitney *U tests* were applied. Categorical variables were analyzed using Chi-Square tests. The association between ABSI quartiles and AAC scores and SAAC was explored using multivariate linear regression and trend tests. Model 1 was not adjusted for any variables, Model 2 was adjusted for age, gender, marital status, race, and education level, and Model 3 further adjusted for alcohol consumption, smoking, diabetes, hypertension, CHD, stroke, cancer, arthritis, COPD, liver condition, total calcium, cholesterol, triglycerides, uric acid, creatinine, and (25(OH)D3) based on Model 2. A smooth curve was plotted to visualize the relationship between ABSI and AAC scores and SAAC. Subgroup analysis and interaction tests were performed by gender (male/female), diabetes (yes/no), and hypertension (yes/no). Considering the complex sampling characteristics of NHANES, sample weights were included in all the above analyses. Receiver Operating Characteristic (ROC) curves were plotted to compare the predictive ability of ABSI, Weight-Adjusted Waist Index (WWI), Body Mass Index (BMI), Waist-to-Height Ratio (WHtR), and Waist Circumference (WC). A p-value < 0.05 was considered statistically significant.

## Results

### Baseline characteristics

The present study included 2741 participants, with weighted percentages of 48.43 % for males and 51.57 % for females. The incidence rate of AAC was 29.12 %, and the incidence rate of SAAC was 7.94 %. The quartiles of ABSI were as follows: Q1 (0.06780‒0.08013), Q2 (0.08013‒0.08302), Q3 (0.08302‒0.08596), and Q4 (0.08596‒0.10795). Table 1 shows the differences in AAC score, AAC, and SAAC across the ABSI quartiles. Compared with the lowest quartile, participants in the highest ABSI quartile had significantly higher AAC scores and a higher prevalence of both AAC and SAAC (*p* < 0.05). Table 2 summarizes demographic and lifestyle characteristics across ABSI quartiles; age, gender, education level, race, alcohol consumption, and smoking all differed significantly across ABSI quartiles (all *p* < 0.05). Table 3 presents clinical comorbidities and laboratory parameters across ABSI quartiles, and significant differences were observed for cholesterol, triglycerides, uric acid, creatinine, 25(OH)D3, diabetes, hypertension, CHD, stroke, cancer, arthritis, COPD, and liver condition.

**Table 1:** AAC score, AAC, and SAAC across ABSI quartiles in the study population.

Variables	Q1 (*n* = 685)	Q2 (*n* = 685)	Q3 (*n* = 685)	Q4 (*n* = 686)	p-value
**AAC scores**	0.75 ± 2.11	1.27 ± 3.06	1.84 ± 3.68	2.77 ± 4.49	<0.0001
**AAC**					<0.0001
Yes	135 (17.83 %)	175 (25.99 %)	223 (32.12 %)	306 (41.38 %)	
No	550 (82.17 %)	510 (74.01 %)	462 (67.88 %)	380 (58.62 %)	
**SAAC**					<0.0001
Yes	20 (1.78 %)	44 (6.04 %)	73 (8.91 %)	115 (15.50 %)	
No	665 (98.22 %)	641 (93.96 %)	612 (91.09 %)	571 (84.50 %)	

ABSI, A Body Shape Index; AAC, Abdominal Aortic Calcification; SAAC, Severe Abdominal Aortic Calcification.

**Table 2:** Demographic and lifestyle characteristics of participants across ABSI quartiles.

Variables	Q1 (*n* = 685)	Q2 (*n* = 685)	Q3 (*n* = 685)	Q4 (*n* = 686)	p-value
**Age**					<0.0001
≤ 60	509 (78.08 %)	444 (70.11 %)	358 (60.48 %)	198 (34.29 %)	
> 60	176 (21.92 %)	241 (29.89 %)	327 (39.52 %)	488 (65.71 %)	
**Gender**					<0.0001
Male	258 (34.73 %)	321 (46.98 %)	361 (56.44 %)	383 (56.39 %)	
Female	427 (65.27 %)	364 (53.02 %)	324 (43.56 %)	303 (43.61 %)	
**Marital status**					0.465
Married	440 (68.64 %)	455 (69.44 %)	444 (71.54 %)	416 (65.82 %)	
Unmarried	245 (31.36 %)	230 (30.56 %)	241 (28.46 %)	270 (34.18 %)	
**Education level**					0.0122
Under high school	107 (9.87 %)	147 (14.62 %)	166 (15.22 %)	176 (18.42)	
High school or equivalent	158 (20.31 %)	155 (21.77 %)	156 (24.47 %)	156 (20.85)	
College graduate or above	420 (69.82 %)	383 (63.61 %)	363 (60.31 %)	354 (60.73)	
**Race**					0.0402
Mexican American	85 (7.18 %)	100 (8.04 %)	93 (6.34 %)	84 (5.53 %)	
Other Hispanic	53 (3.94 %)	79 (5.77 %)	81 (5.8 %)	53 (3.03 %)	
Non-Hispanic	454 (81.71 %)	410 (79.10 %)	418 (80.72 %)	481 (86.12 %)	
Other Race	93 (7.17 %)	96 (7.09 %)	93 (7.14 %)	68 (5.32 %)	
**Alcohol consumption**					0.8958
Yes	498(79.51 %)	482 (78.81 %)	489 (78.43 %)	499 (77.33 %)	
No	187(20.49 %)	203 (21.19 %)	196 (21.57 %)	187 (22.67 %)	
**Smoking**					<0.0001
Yes	246 (33.12 %)	303 (43.17 %)	333 (51.49 %)	380 (54.63 %)	
No	439 (66.88 %)	382 (56.83 %)	352 (48.51 %)	306 (45.37 %)	

ABSI, A Body Shape Index.

**Table 3:** Clinical comorbidities and laboratory parameters of participants across ABSI quartiles.

Variables	Q1 (*n* = 685)	Q2 (*n* = 685)	Q3 (*n* = 685)	Q4 (*n* = 686)	p-value
**Total calcium (mmoL/L)**	2.36±0.10	2.36±0.09	2.37±0.09	2.36±0.09	0.68
**Cholesterol (mmoL/L)**	5.12±1.00	5.11±1.23	5.09±1.14	4.93±1.14	0.004
**Triglycerides (mmoL/L)**	1.50±1.11	1.86±2.85	1.90±1.30	2.00±1.39	<0.0001
**Uric acid (µmoL/L)**	5.23±1.34	5.40±1.37	5.46±1.38	5.73±1.41	<0.0001
**Creatinine (µmoL/L)**	79.16±25.41	79.67±28.74	86.12±51.00	90.64±70.18	<0.0001
**25(OH)D3 (nmoL/L)**	64.68±29.69	64.86±28.04	63.85±27.27	69.50±30.79	0.001
**Diabetes**					<0.0001
Yes	55 (5.72 %)	95 (10.78 %)	133 (16.08 %)	171 (20.18 %)	
No	630 (94.28 %)	590 (89.22 %)	552 (83.92 %)	515 (79.82 %)	
**Hypertension**					<0.0001
Yes	284 (38.76 %)	278 (35.37 %)	332 (46.64 %)	404 (54.43 %)	
No	401 (61.24 %)	407 (64.63 %)	353 (53.36 %)	282 (45.57 %)	
**CHD**					<0.0001
Yes	13(1.5 %)	30 (3.76 %)	40 (5.96 %)	64 (8.72 %)	
No	672 (98.5 %)	655 (96.24 %)	645 (94.04 %)	622 (91.28 %)	
**Stroke**					<0.0001
Yes	18 (2.38 %)	18 (1.67 %)	30 (4.26 %)	50 (5.99 %)	
No	667 (97.62 %)	667 (98.33 %)	655 (95.74 %)	636 (94.01 %)	
**Cancer**					0.0002
Yes	57 (10.5 %)	82 (13.02 %)	93 (18.06 %)	124 (20.69 %)	
No	628 (89.5 %)	603 (86.98 %)	592 (81.94 %)	562 (79.31 %)	
**Arthritis**					0.0077
Yes	189 (30.2 %)	217 (33.71 %)	246 (39.78 %)	299 (41.94 %)	
No	496 (69.8 %)	468 (66.29 %)	439 (60.22 %)	387 (58.06 %)	
**COPD**					<0.0001
Yes	7 (0.76 %)	16 (2.8 %)	31 (5.7 %)	60 (8.65 %)	
No	678 (99.24 %)	669 (97.2 %)	654 (94.3 %)	626 (91.35 %)	
**Liver condition**					0.0289
Yes	24 (2.29 %)	34 (3.77 %)	37 (4.57 %)	42 (5.92 %)	
No	661 (97.71 %)	651 (96.23 %)	648 (95.43 %)	644 (94.08 %)	

ABSI, A Body Shape Index; 25(OH)D3, 25-Hydroxyvitamin D3; CHD, Coronary Heart Disease; COPD, Chronic Obstructive Pulmonary Disease.

### Association between ABSI and AAC scores and SAAC

**Fig. 2:**
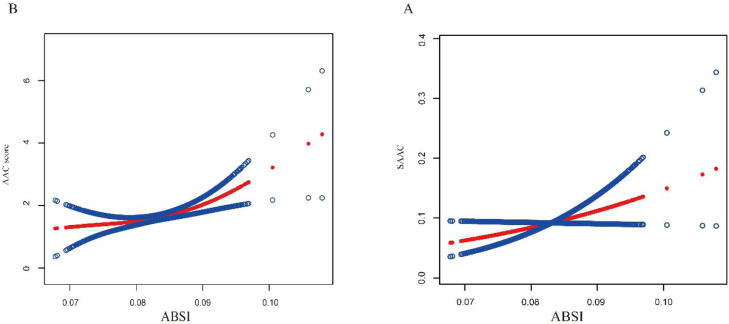
Smooth curves association between ABSI and AAC scores and SAAC. The red line represents the smooth curve fit between ABSI and AAC. Blue line bands represent 95 % Confidence Intervals of the fit. (A) ABSI and AAC score. (B) ABSI and SAAC.

Table 4 reveals the association between ABSI and AAC scores. When ABSI is treated as a continuous variable, there is a positive correlation between ABSI and AAC scores across all models. Specifically, in Model 3, for each one-unit increase in ABSI, the AAC score increases by 0.42-points (95 % CI: 0.29, 0.63). Additionally, AAC scores increased with higher ABSI quartiles in all three models. In Model 3, compared to the first quartile, each 1-unit increase in the highest ABSI quartile was associated with a 0.87-point (95 % CI: 0.49, 1.25) increase in AAC scores. Table 5 shows that ABSI and SAAC were strongly positively correlated in all three models. When ABSI is treated as a continuous variable, in Model 3, each one-unit increase in ABSI is associated with a 37 % (95 % CI: 1.12, 1.77) higher incidence of SAAC. The risk of SAAC increased significantly with higher ABSI quartiles in all three models. In Model 3, the risk of SAAC in the highest ABSI quartile was 1.64 times (95 % CI: 1.56, 4.47) higher than in the lowest quartile. In both adjusted and unadjusted models, the p for trend was < 0.001, suggesting that as ABSI quartiles increased, both AAC scores and the incidence of SAAC notably increased. Smooth curves demonstrated a nonlinear positive correlation between ABSI and both AAC scores and SAAC (Fig. 2).

**Table 4:** Associations between ABSI and AAC scores.

ABSI group	Model 1		Model 2		Model 3	
	β (95 % CI)	p-value	β (95 % CI)	p-value	β (95 % CI)	p-value
Continuous ABSI	0.61 (0.43, 0.86)		0.54 (0.31, 0.72)		0.42 (0.29, 0.63)	
ABSI classification						
Q1	Reference		Reference		Reference	
Q2	0.52 (0.15, 0.88)	0.0053	0.39 (0.04, 0.75)	0.0301	0.31 (−0.03, 0.66)	0.0780
Q3	1.08 (0.72, 1.45)	<0.0001	0.72 (0.36, 1.08)	0.0001	0.56 (0.21, 0.91)	0.0020
Q4	2.02 (1.65, 2.38)	<0.0001	1.17 (0.79, 1.55)	<0.0001	0.87 (0.49, 1.25)	<0.0001
p for trend		<0.0001		<0.0001		<0.0001

Model 1 adjust for: None. Model 2 adjust for: Age, gender, marital status, race, and education level. Model 3 adjust for: Age, gender, marital status, race, education level, alcohol consumption, smoking, diabetes, hypertension, CHD, stroke, cancer, arthritis, COPD, liver condition, total calcium, cholesterol, triglycerides, uric acid, creatinine, and 25(OH)D3.

ABSI, A Body Shape Index; SAAC, Severe Abdominal Aortic Calcification; CHD, Coronary Heart Disease; COPD, Chronic Obstructive Pulmonary Disease; 25(OH)D3, 25-Hydroxyvitamin D3; OR, Odds Ratio.

**Table 5:** Associations between ABSI and SAAC.

ABSI group	Model 1		Model 2		Model 3	
	OR (95 % CI)	p-value	OR (95 % CI)	p-value	OR (95 % CI)	p-value
Continuous ABSI	1.73 (1.42, 2.21)		1.55 (1.29, 1.93)		1.37 (1.12, 1.77)	
ABSI classification						
Q1	Reference		Reference		Reference	
Q2	2.28 (1.33, 3.91)	0.0027	1.99 (1.14, 3.45)	0.0151	1.76 (1.00, 3.10)	0.0507
Q3	3.97 (2.39, 6.58)	<0.0001	2.89 (1.71, 4.88)	<0.0001	2.45 (1.43, 4.20)	0.0011
Q4	6.70 (4.11, 10.91)	<0.0001	3.42 (2.05, 5.70)	<0.0001	2.64 (1.56, 4.47)	0.0003
p for trend		<0.0001		<0.0001		0.0002

Model 1 adjust for: None. Model 2 adjust for: Age, gender, marital status, race, and education level. Model 3 adjust for: Age, gender, marital status, race, education level, alcohol consumption, smoking, diabetes, hypertension, CHD, stroke, cancer, arthritis, COPD, liver condition, total calcium, cholesterol, triglycerides, uric acid, creatinine, and 25(OH)D3.

ABSI, A Body Shape Index; SAAC, Severe Abdominal Aortic Calcification; CHD, Coronary Heart Disease; COPD, Chronic Obstructive Pulmonary Disease; 25(OH)D3, 25-Hydroxyvitamin D3; OR, Odds Ratio.

### Subgroup analysis

**Fig. 3:**
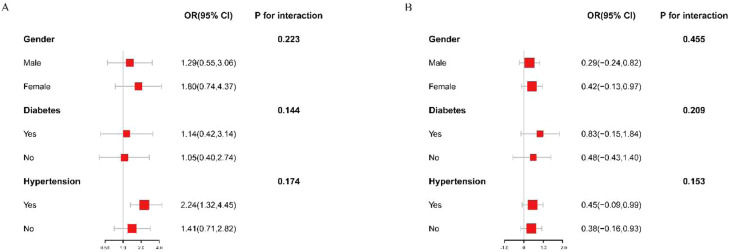
Forest plots of subgroup analysis according to model 3. (A) Subgroup analysis between ABSI and AAC scores. (B) Subgroup analysis between ABSI and SAAC.

To assess the consistency of the nonlinear positive correlation of ABSI with AAC scores and SAAC in the population, the authors performed subgroup analysis and interaction tests by gender (male/female), diabetes (yes/no), and hypertension (yes/no), and only observed a positive correlation between ABSI and AAC scores in the hypertensive population (OR = 2.24, 95 % CI 1.32‒4.45). The overall results of the subgroup analysis showed that the positive association of ABSI with AAC scores and SAAC was independent of gender, diabetes, and hypertension (p for interaction > 0.05) (Fig. 3).

### The predictive ability of the ABSI

**Fig. 4:**
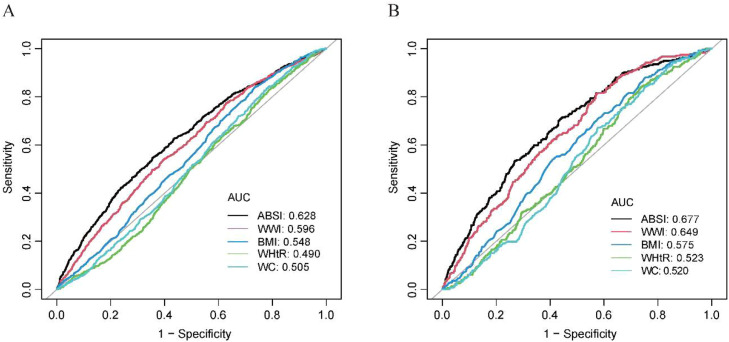
ABSI, WWI, BMI, WHtR, and WC predict ROC curves for AAC and SAAC. (A) ROC curve in AAC. (B) ROC curve in SAAC.

The ROC curves showed that the Area Under the Curves (AUCs) of ABSI, WWI, BMI, WHtR, and WC were 0.628, 0.596, 0.548, 0.490, and 0.505 in predicting AAC, and 0.677, 0.649, 0.575, 0.523, and 0.520 in predicting SAAC (Fig. 4). ABSI had the highest AUC for both predictions, indicating that ABSI has valuable predictive ability.

## Discussion

Through a weighted analysis of 2741 participants, the present study found that ABSI was positively correlated with AAC scores and SAAC after fully adjusting for covariates, suggesting that individuals with higher ABSI are more likely to develop AAC and SAAC. Additionally, ROC analysis revealed that AISI had stronger predictive ability for AAC and SAAC compared to WWI, BMI, WHtR, and WC.

Previous studies have shown a close correlation between abdominal fat accumulation and AAC.^16^, ^17^, ^18^ Through analysis of abdominopelvic CT in 302 participants, Ivan et al. reported that AAC was significantly and positively correlated with the amount of visceral adipose tissue and that the abdominal aorta was the most calcified vessel compared to other abdominal vessels (6). A prospective study showed that visceral obesity was independently associated with the severity of AAC (OR = 6.63, 95 % CI 1.90‒23.14), indicating that visceral obesity may have a contributory effect on AAC.^19^ Cross-sectional studies have found that visceral obesity is positively associated with both AAC scores and SAAC (β = 0.04, 95 % CI 0.01‒0.08; OR = 1.04, 95 % CI 1.01‒1.07) and that this correlation is independent of sex, age, BMI, hypertension, and diabetes.^20^ Compared to the disadvantage of BMI, which does not distinguish between muscle and adipose tissue, the ABSI calculation takes into account the relationship between height, weight, and waist circumference and therefore better reflects the amount of abdominal fat accumulation in individuals.^10^^,^^21^ The present study showed that the ability of ABSI to predict AAC and SAAC was better than that of WWI, BMI, WHtR, and WC, which is consistent with other studies. Existing studies have indicated that ABSI is a better predictor of diabetes, cardiovascular disease, hypertension, and all-cause mortality than BMI.^11^^,^^22^, ^23^, ^24^ Ning et al. reported that the change in ABSI was associated with mortality among people aged 65-years or older in China, with each 10 % increase in ABSI increasing the risk of death in the study population by 1.9 %.^25^ In the present study, after fully adjusting covariates, ABSI still shows a strong positive correlation with AAC score and SAAC, and the smooth curves visualize this relationship, suggesting that ABSI is a valuable predictor of AAC and SAAC.

In addition, the subgroup analysis indicated that the positive association between ABSI and AAC was more pronounced among individuals with hypertension. This is consistent with prior evidence that the occurrence of AAC in hypertensive patients is approximately 1.66-times that of non-hypertensive individuals (OR = 1.66, 95 % CI 1.30–2.13), suggesting that elevated blood pressure may amplify the impact of abdominal adiposity on vascular calcification.^26^ Although the underlying mechanism is not yet fully understood, it has been proposed that long-term hypertension may promote AAC by inducing hemodynamic stress and endothelial injury in the aortic wall, thereby accelerating vascular remodeling and calcific degeneration.^27^

Fewer studies have been conducted to investigate the mechanisms of abdominal obesity and AAC. Adipose tissue has been reported to secrete adiponectin, leptin, Tumor Necrosis Factor-alpha (TNF-a), and Interleukin-6 (IL-6), which can cause insulin resistance, endothelial dysfunction, and promote arterial calcification.^28^^,^^29^ Panagiotis et al. reported that adipose tissue secretes adipokines leptin, adiponectin, and other adipokines, of which leptin has a thrombogenic effect that promotes vascular inflammation and increases oxidative stress through leptin receptors, a process that can lead to the formation of arterial calcification, and adiponectin can inhibit the macrophages into foam cells and reduce their phagocytic activity, causing arterial calcification.^30^ However, the exact mechanism by which abdominal fat leads to AAC calcification is not fully understood due to a lack of research; further studies are needed to elucidate it.

Although several studies have investigated the relationship between ABSI and AAC, the existing literature still has notable limitations. For example, most studies did not examine the association between ABSI and SAAC, were based on relatively small sample sizes, and did not incorporate sampling weights to achieve population-level representativeness. In this context, the present study advances the field in several ways. Using data from NHANES 2013–2014, the authors evaluated the relationship between ABSI and both AAC scores and SAAC in a nationally representative U.S. adult population. The authors further visualized the dose–response pattern between ABSI and AAC/SAAC using smooth curves, and the authors conducted subgroup analyses to explore the stability of this association across clinically relevant strata. These strengths enhance the interpretability and relevance of the present findings. This study also has some limitations: First, NHANES only assessed AAC in participants aged 40-years and older, so individuals under 40-years of age were not included. Therefore, it is unclear whether these findings can be generalized to younger populations. Second, because this is not a longitudinal cohort study, the authors cannot establish a causal relationship between ABSI and AAC/SAAC. Prospective studies are needed to determine temporality and to evaluate whether changes in ABSI over time correspond to the progression of aortic calcification.

The strong association between ABSI and AAC/SAAC suggests that this easily calculated index could be integrated into public health strategies for primary prevention. In clinical and community settings, ABSI could serve as a simple, low-cost tool to identify individuals with high central adiposity who may be at increased risk of subclinical vascular disease. Early identification of these high-risk individuals would allow timely and targeted lifestyle interventions, such as promoting physical activity, weight management, and dietary patterns that reduce visceral fat, with the goal of preventing or delaying vascular calcification and subsequent cardiovascular complications at the population level.

## Conclusions

The present findings suggest that ABSI is nonlinearly and positively related to AAC scores and SAAC, indicating its potential predictive value for both conditions. However, future longitudinal studies are needed to confirm these relationships.

## Availability of data and materials

All data in this study can be accessed by visiting the official NHANES website: https://www.cdc.gov/nchs/nhanes/.

## Ethics approval and consent to participate

NHANES was approved by the Ethics Review Board of the NCHS (Protocol n° 2011–17). The study was performed in accordance with the principles of the Declaration of Helsinki. Participants provided written informed consent.

## Authors’ contributions

Youfu Wang: Conceptualization; formal analysis; methodology; software; writing-original draft.

Yiming Su: Data curation; formal analysis; validation; writing-original draft.

Changzhi Luo: Data curation; formal analysis; software; writing-original draft.

Han Yang: Data curation; methodology; writing-review & editing.

Xiao Qin: Funding acquisition; resources; project administration; supervision; writing-original draft; writing-review & editing.

## Funding

This work was supported by the National Natural Science Foundation of China: 81960091, and Prof. Xiao Qin from the First Affiliated Hospital of Guangxi Medical University is the recipient.

## Data availability

The datasets generated and/or analyzed during the current study are available from the corresponding author upon reasonable request.

## Conflicts of interest

The authors declare no conflicts of interest.
